# Temperature Modulation of Electric Fields in Biological Matter

**DOI:** 10.1371/journal.pone.0020877

**Published:** 2011-06-13

**Authors:** Charlotte S. Daniels, Boris Rubinsky

**Affiliations:** 1 Department of Mechanical Engineering, University of California, Berkeley, California, United States of America; 2 Graduate Program in Biophysics, University of California, Berkeley, California, United States of America; University of South Florida College of Medicine, United States of America

## Abstract

Pulsed electric fields (PEF) have become an important minimally invasive surgical technology for various applications including genetic engineering, electrochemotherapy and tissue ablation. This study explores the hypothesis that temperature dependent electrical parameters of tissue can be used to modulate the outcome of PEF protocols, providing a new means for controlling and optimizing this minimally invasive surgical procedure. This study investigates two different applications of cooling temperatures applied during PEF. The first case utilizes an electrode which simultaneously delivers pulsed electric fields and cooling temperatures. The subsequent results demonstrate that changes in electrical properties due to temperature produced by this configuration can substantially magnify and confine the electric fields in the cooled regions while almost eliminating electric fields in surrounding regions. This method can be used to increase precision in the PEF procedure, and eliminate muscle contractions and damage to adjacent tissues. The second configuration considered introduces a third probe that is not electrically active and only applies cooling boundary conditions. This second study demonstrates that in this probe configuration the temperature induced changes in electrical properties of tissue substantially reduce the electric fields in the cooled regions. This novel treatment can potentially be used to protect sensitive tissues from the effect of the PEF. Perhaps the most important conclusion of this investigation is that temperature is a powerful and accessible mechanism to modulate and control electric fields in biological tissues and can therefore be used to optimize and control PEF treatments.

## Introduction

The current direction of surgical technologies is toward a minimally and non-invasive strategy. In comparison to traditional surgery, minimally and non-invasive surgeries are positioned to transform the field of medicine with shorter hospital stays, reduced surgical trauma, improved immune response and greater precision [1]. These benefits are primarily due to less intrusive procedures and exceptionally targeted tissue treatment.

Pulsed electric fields (PEF) are becoming commonly used in minimally invasive and non-invasive surgery. The PEF technologies utilize electric fields which target the cellular membrane, increasing membrane permeability through the formation of nanoscale defects. The PEFs are usually delivered through electrodes in contact with the tissue. Typical PEF parameters currently used in medicine and biotechnology employ 0.1 to 1E7V/m electric fields, nanosecond to millisecond pulse lengths and one to several hundred pulses. They can be AC or DC. PEFs can have two different effects on the cellular membrane as a function of the electrical parameters: reversible and irreversible electroporation. Reversible electroporation causes the transient permeabilization of the cell membrane. Reversible electroporation is used in genetic engineering for the introduction of genes into targeted cells [2], [3], [4]. It is also used for tissue ablation in electrochemotherapy, which introduces drugs such as bleomycin into PEF treated cells [5], [6], [7]. In irreversible electroporation (IRE), the effect of membrane permeabilization leads to cell death. In the past, it was thought that IRE was produced by microsecond to millisecond pulses only. However, it was recently found that nanosecond pulses also produce IRE when used with appropriate electric field strength [8], [9]. During the last four decades, IRE was used primarily in the food industry for sterilization of micro-organisms [10], [11]. Recently, IRE has emerged as an important minimally invasive technique for tissue ablation, because of its molecular selectivity [12],[13].

Mathematical studies have shown that naturally occurring local heterogeneities in the electrical properties of tissue affect the applied electric fields during PEF and thereby affect the outcome of PEF procedures [14],[15]. It is well established that temperature affects the electrical properties of tissue [16]. It occurred to us that temperature could be used to locally modify electrical tissue properties in a desirable and controlled way and thereby produce greater local control over the outcome of minimally invasive PEF procedures. The goal of this study is, therefore, to explore the hypothesis that local changes in electrical properties of tissue produced by changes in temperature can be used to modulate and control the effect of PEFs on biological matter. This is a mathematical study using the numerical analysis tools described in [14].

Electrical conductivity of tissue is a function of temperature, exhibiting a positive correlation. Consequently, the electrical conductivity of cooled tissues is substantially lower than those at physiological temperatures. In this study, we will focus on an examination of the effect of lowering the temperature locally on the outcome of the PEF protocol. While many methods for cooling tissue locally are possible, this study will simulate the use of cooling probes. Such probes are commonly used in minimally invasive surgery at temperatures below freezing (Rubinsky 2000). Here it is assumed that for this particular application, the probes are cooled to above freezing temperatures and used only as a means for changing tissue electrical properties.

The biophysical effect of temperature on the process of electroporation will be also considered and incorporated into the mathematical model. Several studies have investigated the effects of temperature on electroporation. For instance, Diaz investigated the effect of low temperatures on electroporation efficacy [17]. He accomplished electroporation on kidney epithelial cells at temperatures as low as −2°C [18]. Additionally, Gallo demonstrated the trend between temperature and electroporation; as temperature decreases, initial cell membrane permeabilizing pulse voltage increases [19]. Gallo's study operated on the stratum corneum in the range 0–80°C. The same relationship between temperature and electroporation was found in *alga Valonia*, rye leaf protoplast and mammalian cell lines [20].

As a first order study, the concept introduced here investigated a mathematical analysis of temperature and electric fields produced by the application of PEFs in conjunction with local cooling. The focus of this theoretical study is to examine the effect of changes in temperature on electric fields and the subsequent implications for tissue electroporation. For simplicity and to extract the most salient biophysical aspects of the analysis, we investigate simple two dimensional configurations in cylindrical coordinates in which the electrical and thermal effects are produced by electroporation probes and cooling probes with dimensions typical to commercially available devices. This study focuses on the change in electrical parameters as a result of temperature. It is evident that following this first order analysis, much additional theoretical and experimental work remains to be done.

## Methods

The models were generated using numerical analysis executed by Comsol Multiphysics (version 4.1). This initial study utilized 2-dimensional models because these are sufficient to demonstrate the significant effect of temperature induced electrical property heterogeneities. Two equations were solved simultaneously in Comsol. The first of which was the Laplace equation for potential distribution associated with an electric pulse:
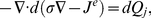
(1)where *σ* is electrical conductivity, *V* is voltage, *J^e^* is external current density, *d* is thickness and *Q _j_* is the current source. For all boundaries, the external current density and the current source were set to zero, and thickness was set to one. The electric field was solved in the AC/DC Conductive Media module using a transient analysis to account for electrical pulses.

The electrical conductivity, *σ*, was determined by the local tissue temperature according to data from the literature. Properties for physiological saline solution were used as a first order simulation for biological tissue, since electrical property data for tissues in the entire range of temperatures of interest is not available. Data from J.J. Arps [21] was curve fitted to calculate the electrical conductivity of the composite medium. The derived electrical conductivity also employed experimental data from [22], resulting in the following function:
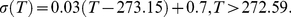
(2)Equation 2 describes the behavior of electrical conductivity in [S/m] as a function of temperature *T* in [K]. The correlation coefficient of this equation relative to experimental data was tabulated to be *r = 0.99989*.

In addition to electrical conductivity, electrical permittivity is also a function of temperature. The following equation [23] was utilized to take into account the temperature dependence of electrical permittivity:
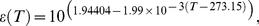
(3)where *ε* is electrical permittivity and *T* is temperature in [K]. This equation is valid for low frequency permittivity, experienced by typical PEF pulse parameters, which are in the range of 0.1-20E-3 seconds [24], [25].

The temperature distribution was obtained from the solution of the Pennes bioheat equation, which was solved simultaneously as the electrical potential equation. The general Pennes bioheat equation to be solved for this case took the following form:
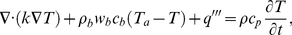
(4)where *k* is thermal conductivity, *T* is temperature, *w_b_* is blood perfusion, *c_b_* is the heat capacity of blood, *T_a_* is arterial temperature, *ρ* is the tissue density, *c_p_* is the tissue heat capacity and 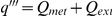. *Q_met_* is the metabolic heat generation and 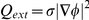, which accounts for Joule heating, where 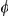 is electrical potential and *σ* is electrical conductivity of the tissue. In this study, the effect of Joule heating on the temperature distribution was considered. The values for biological tissue utilized in the Pennes bioheat equation are listed in Table 1 [26].

**Table 1:** Temperature dependent properties used in the Pennes bioheat equation for the purpose of solving the temperature distribution due to the application of the cooling probe.

Thermal Conductivity	Blood Perfusion	Blood Heat Capacity	Metabolic Heat	Tissue Density	Tissue Heat Capacity
0.5 [W/mK]	0.5[kg/m^3^ s]	3640 [J/kg K]	33800[W/m^3^]	1000 [kg/m^3^]	3750 [J/kgK]

**Figure 1:**
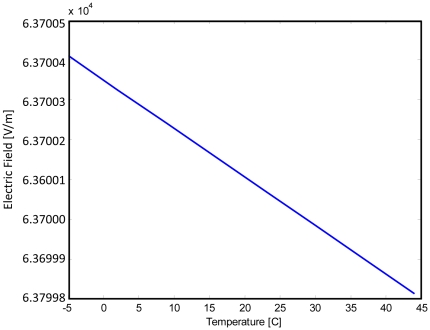
This plot depicts the threshold electric field for irreversible electroporation as a function of temperature. It is clear that as temperature is decreased, the required electric field to achieve electroporation increases. However, the effect of temperature on electric field threshold is very small, on the order of 0.1 V/m.

Studies on the effect of temperature on electroporation protocols have revealed a negative correlation between temperature and fields. The electric fields required for producing electroporation increase as temperature decreases. The goal of this study is to investigate the ultimate effects of temperature modulation on PEF protocols, such as the fields necessary to induce reversible and irreversible electroporation. To accomplish this, data has been extracted from existing literature to produce a correlation between temperature and the fields required for reversible and irreversible electroporation [27]. The equation used in this study was derived from the experimental data acquired in [17] and is given by:
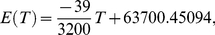
(5)where *T* is temperature [°C] and *E* is electric field [V/m]. Equation 5 is plotted in Figure 1. It is clear from both the equation and the graph that temperature dependence of the electric field is very low. However, the data available is very limited and cells in tissue may behave differently from cells in solution. For this reason, this equation was used as an approximate correlation for this study. It was used primarily to demonstrate an accurate methodology, but a substantial amount of additional research is needed to generate precise correlations, which currently have not yet been developed.

### Models

The primary goal of this study is to examine how local changes in tissue electrical properties due to changes in temperature affect electric fields during PEF protocols. This study will focus in particular on the effects of cold temperatures. The complex volumetric effect of temperature induced changes in tissue electrical properties can be approximated by voltage divider circuits consisting of elements of resistance in series or in parallel. To capture these effects two models were examined. Case 1 includes temperature induced resistance in series with the original tissue impedance. Case 2 includes temperature induced resistance in parallel. These two cases can also be viewed as the difference between an electrically active cooling probe (resistance in series) and an electrically inactive cooling probe (resistance in parallel). These descriptions are only an approximation of the more complex models investigated in this study, and are described here for clarification of the resulting electrical phenomena.

#### Case 1: Resistors in Series

The first geometry considered was represented in 2D and consisted of a single cryoprobe inserted into the center of a sample of tissue. The cryoprobe simultaneously applied a cold temperature and voltage pulses. In this case, the cooling of tissue simulates the addition of a resistor in series. The cryoprobe, measuring 3.4 mm in diameter [28], was inserted into the center of an infinitely long cylinder of tissue, 6 cm in radius. The outer edge of the cylinder was set to constant deep body temperature, 37°C. The initial temperature of the system was also deep body temperature.

Cryoprobes are designed as hollow conduits through which a coolant flows. Because the probes are made of electrically conductive metal, the same probe can be used to both cool the tissue and apply PEFs as electrodes when connected to a voltage supply. Therefore, the cold probe was modeled as a PEF electrode. A typical PEF voltage of 2500 V was applied between the cold probe and the uniformly grounded outer edge of the tissue cylinder. A typical irreversible electroporation pulse sequence of 50 µs pulse length at a frequency of 1 Hz was used. This geometry was chosen because it simulates a typical clinical procedure during which a single probe applies a voltage and a grounding pad is placed at a distance, similar to radio-frequency ablation technique.

To understand the implications of temperature induced effects on the electric field, two variations of Case 1 have been modeled. The first applies a temperature of 0°C to the cold probe. The second applies a thermally insulating boundary condition to the probe, which serves as a control.

**Figure 2:**
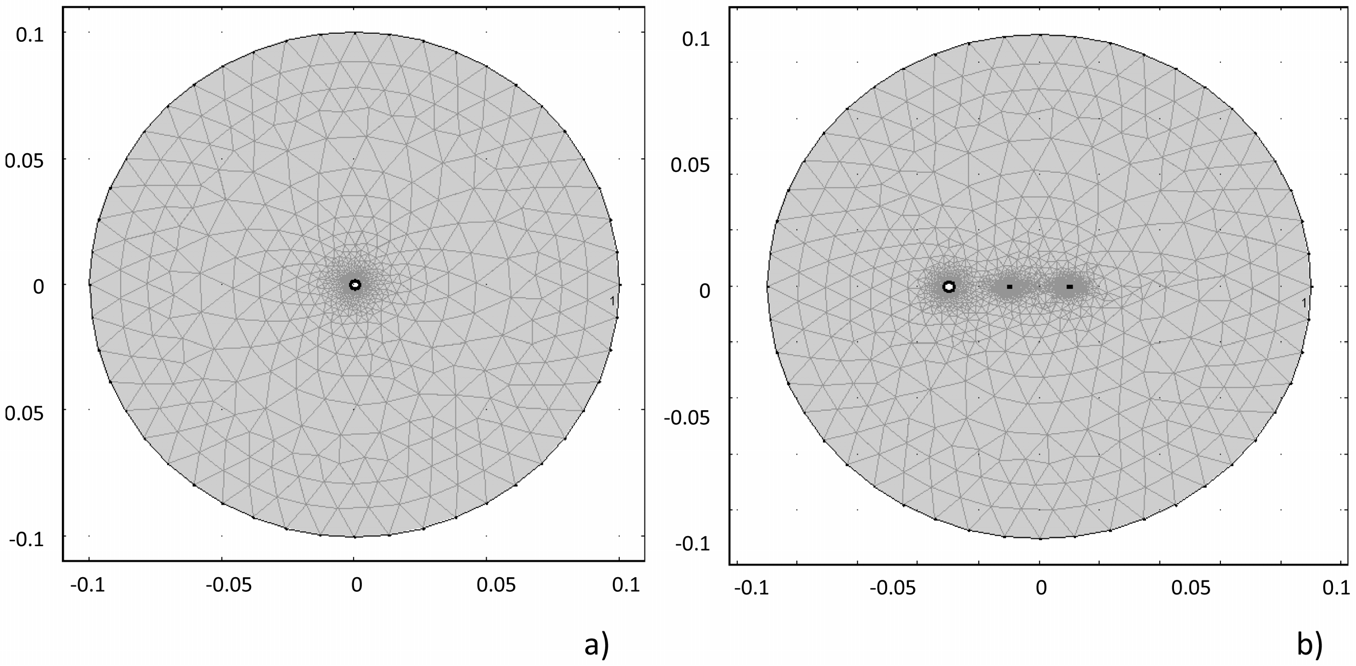
The finite element mesh for the two geometries investigated in this study. a) Mesh for Case 1, in which one cold probe is utilized for both cooling and as a PEF electrode. b) Mesh for Case 2, in which two typical electroporation probes are used to deliver the electric pulses and one cold probe is used to cool the tissue and induce local changes in electric properties. Note that the mesh is extra fine in the vicinity of the probes in order to capture the changes in electric field and temperature that occur due to the effect of the probes.

The finite element mesh for this case utilized triangular elements, as shown in Figure 2a. The element size was smallest adjacent to the cryoprobe, and increased in size as it radiated towards the outer boundary. This was done in order to accurately capture the steep temperature gradient adjacent to the cold probe. The mesh was refined until the solution was no longer affected. Approximately 3800 elements were utilized to cover a 314 cm^2^ surface area.

#### Case 2: Resistors in parallel

The second 2D geometry utilized three probes: two electrodes and a cooling probe at a distance. Because a single probe is not acting as an electrode and a cooling probe simultaneously, this configuration approximates two resistors in parallel. An infinitely long cylinder of tissue 6 cm in radius was set to constant deep body temperature and electrical insulation at the outer margin. The initial temperature of the system was also deep body temperature. The delivery of PEF was applied through two typical irreversible electroporation electrodes [13] of 1 mm in diameter. The leftmost electrode was held at ground and the rightmost probe applied a voltage of 2500 V. A typical irreversible electroporation pulse sequence of 50 µs pulse length at a frequency of 1 Hz was used. As in Case 1, the cold probe was 3.4 mm in diameter. A temperature of 0°C was applied to the cold probe and it was considered electrically insulated. The cold probe was set at various distances from the electrodes, to study the dependence of temperature induced heterogeneities on geometry. The exact geometries are specified in the Results and Discussion section.

To understand the implications of temperature induced effects on the electric field, two variations of Case 2 have been modeled. The first applies a temperature of 0°C to the cold probe. The second applies a thermally insulating boundary condition to the probe, which serves as a control.

The finite element mesh in this study utilized triangular elements, demonstrated by Figure 2b. The element size was smallest in the region surrounding both probes. This was done in order to accurately capture the temperature gradients adjacent to both of the probes. The mesh was refined until the solution was no longer affected. Approximately 5100 elements were utilized to cover a 314 cm^2^ surface area.

## Results and Discussion

### Case 1: Resistance in Series

The first case investigated was a 2D model with the boundary conditions previously specified. The purpose of this model was to illustrate the temperature induced effects on electric field due to a probe simultaneously delivering PEF and cold temperatures.

#### Cooling

**Figure 3:**
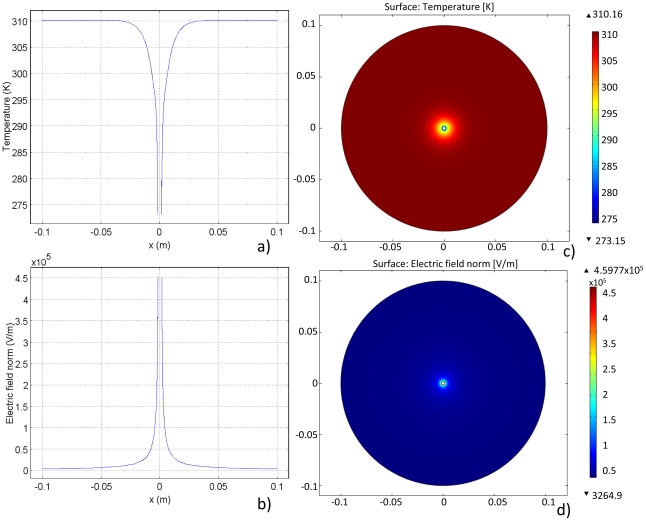
Results for Case 1. a) and b) Temperature and electric field distribution, respectively, at a transection along the diameter of the domain. c) and d) Temperature and electric field surface plots, respectively, in the domain. Plots reflect results after 2000 seconds of cooling at 0°C followed by ten 2500 V pulses (1 Hz, 50 µs) with full bioheat parameters considered.

The temperature distribution after the cooling and PEF procedure is illustrated in Figure 3a. The temperature distribution, as expected, increases from the low temperatures at the cold probe surface to body temperature at the outer boundary. The corresponding electric field is demonstrated in Figure 3b. It is clear from these two graphs that the electric field and temperature distribution are inversely proportional. This relationship is a result of the temperature dependence of electrical conductivity, demonstrated by Equation 2. As a result, the highest electric fields occur in the regions of lowest temperature. It can be seen clearly that the electric field is confined within the low temperature region, and reaches zero nearly everywhere else.

#### Control

**Figure 4:**
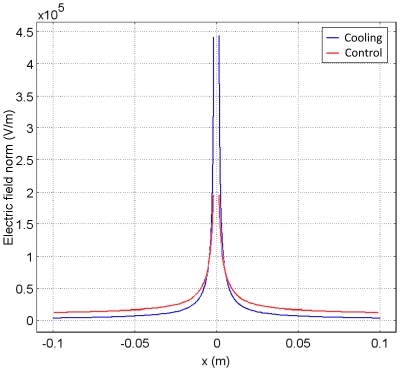
Control results for Case 1. Comparison of electric field distributions along a transection at the diameter of the sample. Blue line: electric field after 2000 seconds of cooling at 0°C followed by ten 2500 V pulses (1 Hz, 50 µs). Red line: control electric field after ten 2500 V pulses (1 Hz, 5 µs), With full bioheat parameters considered. It is clear from this figure that the cooling case achieves a peak electric field of over twice the magnitude of the control case, demonstrating the ability of low temperatures to concentrate and magnify the electric field in this configuration (resistance in series). Additionally, at the outer boundaries of the geometry, it can be seen that the electric field is lower in the cooling case than the control case, which demonstrates the ability of low temperatures to confine the electric field to regions of low temperature in this geometry.

It is pertinent that the electric field produced during cooling be compared to a control study. The control study applied the same electrical boundary conditions as the cooling case to tissue held at body temperature. Figure 4 plots the electric field in the control and cooling case on the same axes for comparison purposes. It is evident from this graph that the electric field in the cooled regions is substantially higher than the field produced in the control study in the same region. The peak electric field in the cooling case is, in fact, three times larger than the control case. However, at a distance from the cooled region, in the location of normal body temperatures, the fields for the cooled case are lower than those in the control. This indicates that cold temperatures are capable of both magnifying the electric field and confining it to the cold regions.

The effects of cooling found in Figures 2 and 3 are caused by the change in the electrical properties of tissue due to temperature. Lower temperatures yield a lower ionic conductivity. The electric field distribution (Figure 4), demonstrates the inversely proportional relationship between temperature and electrical conductivity, as described by Equation 2. For the same voltage boundary conditions, from continuity of ionic current, the electric field will be higher in the regions of lower electrical conductivity. Because of the increased electrical resistance in the regions of colder tissue, in a situation in which the cold region acts as a resistor in series with the area of tissue that is not affected by cooling, the highest electric fields are confined to the cold regions. The fields beyond the cooled regions, in tissue at normal temperature, are substantially lower than those in the cooled regions.

**Figure 5:**
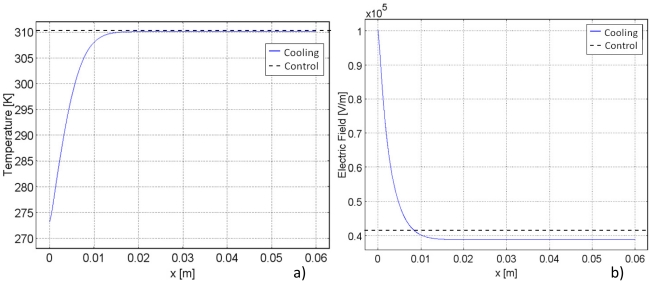
Comparison of cooling and control models for a supplemental one-dimensional study. Graphs illustrate: a) Temperature and b) electric field. Control models are held at constant body temperature.

This effect is seen best in a supplemental analysis, shown in Figure 5. It consists of a simple one-dimensional Cartesian study of a 6 cm slab of tissue between two parallel plates. The leftmost plate applies a voltage of 2500 V and the rightmost plate applies ground. Figure 5 compares the electric field at constant temperature with the case in which cooling at 0°C is applied on the leftmost plate and body temperature, 37°C, is applied to the rightmost plate for 90 seconds. The effect of temperature induced tissue property heterogeneities is evident. At a constant temperature, the electric field is constant across the slab. For the case of cooling at one plate, the field is substantially higher near the cold surface and it decays to low values away from the cold surface. The difference between the electric field in cold regions and the electric field at body temperature is an order of magnitude. These results, as well as those demonstrated in Figures 2 and 3, suggest that inducing heterogeneity in tissue electrical properties through local cooling, with an analogue circuit of two resistors in series, has the effect of confining and enhancing the electric fields in the colder regions and reducing the field in the higher temperature regions.

The results from Case 1 suggest that it should be possible to design PEF protocols in which the PEF induced effects on cells are confined to the cooled regions of tissue and do not extend beyond the cooled regions. Furthermore, cooling the PEF electrode will yield higher electric fields in the vicinity of the probe. The complementary effect of this observation is that the electric fields beyond the cooled area will be substantially reduced. Several recent studies have shown that stray electric fields beyond the PEF treated areas could have negative effects on other organs, such as the heart [29], or create undesirable muscle contractions. Reducing the electric fields beyond the treated area with cold should also reduce these effects. In addition, it is well established that the electric currents in PEF treatment of tissues are very high, on the order of tens of Amperes. The increased resistance caused by the cooled PEF probes, in a configuration such as the one discussed here, will substantially reduce the currents for the same applied voltage, while, on the other hand increasing the field in the cooled volume.

Note, however, that the effects discussed here, for Case 1, are restricted to situations in which the effect of cooling is that of a resistance in series. The second half of the study will address a situation in which the cooled area is not produced by the PEF probes but rather by a different cooling probe. In that case the effect is that of adding a high resistance in parallel.

### Resistance in Parallel

**Figure 6:**
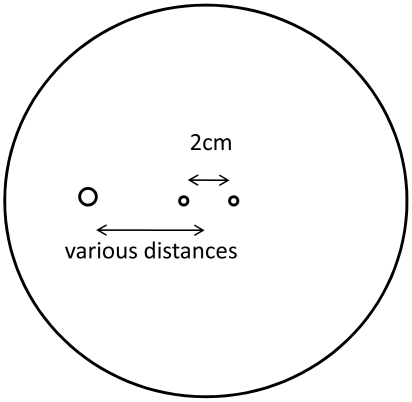
Schematic of the positioning of the cooling probe and electrodes in the different variations of Case 2.

The configuration examined in the second half of this study is illustrated schematically in Figure 6. In Case 2, the effect of cooling delivered by a probe that is not electrically active on a typical irreversible electroporation protocol is investigated. The PEFs were delivered by two electrodes of 1 mm diameter, separated by 2 cm. The 3.4 mm diameter cold probe was placed at various locations from the center along the axial line connecting the centers of the PEF electrodes. The protocol consisted of 2000 seconds of cooling applied by the cold probe at 0°C, followed by ten, 2500 V pulses (1 Hz, 50 µs length) applied by the PEF electrodes. The cooling probe was electrically insulated. In the control case, ten 2500 V pulses (1 Hz, 50 µs length) applied by the PEF electrodes without using the cooling probe. Full bioheat parameters were utilized to simulate tissue.

**Figure 7:**
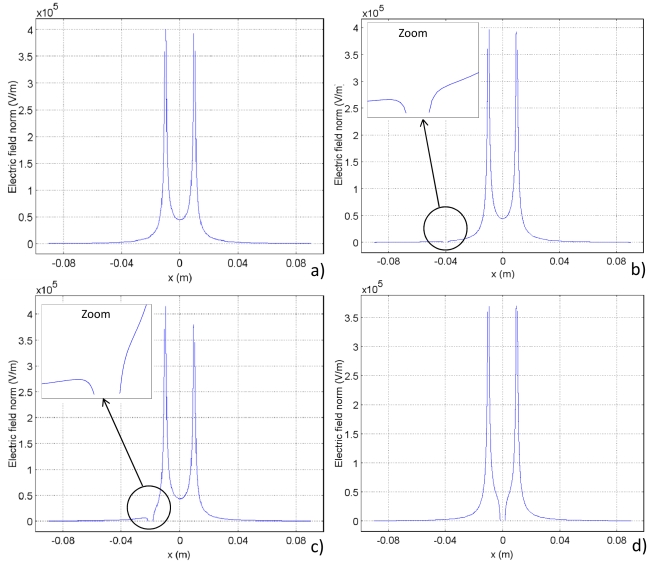
The electric field distributions along a transection at the diameter of the sample for Case 2. a) Control, b) cold probe 4 cm from center, c) cold probe 2 cm from center, and d) cold probe 0 cm from center. These images indicate that in this configuration, resistance in parallel, cooling can be used to protect particular regions in the domain from electric fields. By comparing Figure 7 b, c and d to Figure 7a, it is clear that the cooling temperatures applied lower the electric field in the region of low temperature in this geometry.

Figure 7 illustrates the electric field along a transection through the center of the electrodes and cooling probe for various locations of the cooling probe. Figure 7a shows the control case and Figure 7b shows the cooling case at 4 cm from the center of the domain. It is clear that in the configuration described here the increased electrical resistance due to the cold region acts as a current path of high electrical resistance in parallel to the higher temperature path of lower resistance. This configuration, in contrast to the trends identified in Case 1, results in a substantial decrease in the electric field in the cooled region. In fact, the electric field reaches 0 V/m in the location of the cold probe.

**Figure 8:**
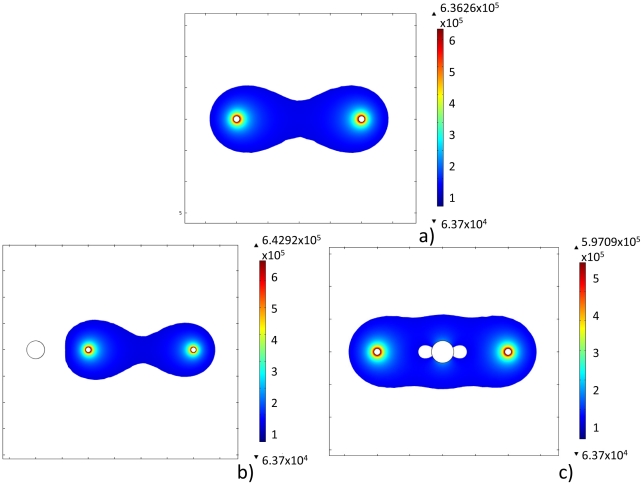
Area of tissue that undergoes irreversible electroporation for Case 2. a) Control case without any cooling applied, b) cooling probe located 2 cm from the center, c) cooling probe located at the center of the tissue.

Figure 8 illustrates a potential application of this observation. Equation 5 has been utilized to calculate the regions of tissue that undergo irreversible electroporation. The results are presented as surface plots in Figure 8. Figure 8 demonstrates that a cooling probe can avoid irreversible electroporation damage at a particular location. Figure 8b demonstrates that this can be accomplished outside of the treatment region when the cooling probe is placed at a distance from the electrodes. Figure 8c demonstrates that a cold probe can protect a region between the two electrodes as well.

These results illustrate a potential important application of the use of cooling in PEFs. When comparing Figure 8a and 7b, it is seen that in 7b the temperature induced changes in electrical potential due to the cold probe cause the irreversible electroporation field near the probe to recede (the edge flattens). This suggests that if a sensitive tissue structure is close to the outer edge of the PEF fields of treatment, the simple application of cold can protect this structure. Figure 8c shows that the use of cold can eliminate PEFs even in the center of the treated area. This suggests that critical tissues, which need protection during a PEF protocol, can be protected by cold. One clinically relevant example is the bladder sphincter, which is particularly vulnerable during the IRE treatment of the prostate. Cooling the sphincter, and thereby increasing its electrical resistance, could protect it from damage during minimally invasive treatment of the prostate with IRE. By increasing the local electrical resistance through the placement of cooling instruments, electric fields can be modulated to obtain desired effects.

### Conclusions

The goal of this study was to evaluate the feasibility and the characteristics of a minimally invasive surgical procedure in which changes in electric properties due to local changes in temperature were used to modulate electric fields during PEF procedures. The hope was that control over the outcome of the procedure will improve as a result. This investigation examined two different possible configurations. The first configuration, Case 1, utilizes the PEF electrode also as a cooling probe, with the resulting electrical effect of a resistance in series. In this configuration, the electric fields are confined to the cold region and magnified in that region while the fields at a distance are reduced. The second configuration, Case 2, applies cold with an element that is not electrically active, with the resulting electrical effect of resistances in parallel. In Case 2, the electric field in the cold region is reduced.

Perhaps the most important conclusion from this study is that temperatures can be used to modulate and control electric fields. Through proper placement of cold, various desirable and controlled PEF protocols can be accomplished that could not have been achieved otherwise. It is clear that a variety of configurations of cold and heat could be used to achieve various PEF goals.

Although this is a first order theoretical analysis and additional theoretical and experimental research is needed to further develop this new concept, these primary results act as a promising foundation for future work. For these reasons, this numerical study has indicated the undeveloped potential and the motivation for pursuing the use of temperature to modulate and control PEF.
